# Protocorm-Supporting Fungi Are Retained in Roots of Mature *Tipularia discolor* Orchids as Mycorrhizal Fungal Diversity Increases

**DOI:** 10.3390/plants10061251

**Published:** 2021-06-20

**Authors:** Melissa McCormick, Robert Burnett, Dennis Whigham

**Affiliations:** Smithsonian Environmental Research Center, Edgewater, MD 21037, USA; burnettr1@gmail.com (R.B.); whighamd@si.edu (D.W.)

**Keywords:** Orchidaceae, orchid mycorrhizae, protocorm fungi, mycorrhizal diversity

## Abstract

Mycorrhizal fungi are critical to understanding the distribution patterns of many plants, but they are especially important for orchids. Some orchids may change the mycorrhizal fungi they use through their lives, either in response to changes in abiotic or biotic conditions, or as a result of ontogenetic changes that alter the orchid’s need for fungal nutrition. The temperate terrestrial orchid *Tipularia discolor* germinates only on decomposing wood, but often persists well after the wood has completely decomposed and has been incorporated into the soil. We used PCR and Sanger sequencing to ask: (1) Do mature *T. discolor* retain protocorm fungi or are protocorm and adult mycorrhizal fungi mutually exclusive? (2) Are protocorm fungi limited to areas with decomposing wood? (3) Does the abundance of protocorm fungi in the substrate differ between decomposing wood and bare soil? We found that *T. discolor* retained protocorm fungi into maturity, regardless of whether they were growing in persistent decomposing wood or soil. Protocorm fungi were not restricted to decomposing wood but were more common and abundant in it. We conclude that the mycorrhizal fungi associated with *T. discolor* change during the ontogeny of individuals. These results highlight the importance of assessing protocorm fungi, in addition to mycorrhizal fungi associating with adult orchids, to understand the conditions needed for orchid germination, growth, and reproduction.

## 1. Introduction

The Orchidaceae is one of the most species-rich and diverse families of flowering plants [1]. Orchids occur in terrestrial, wetland, and aerial (epiphytic) habitats and may be photosynthetic, mycoheterotrophic, or varying degrees of both [2,3]. A unifying characteristic of all orchids is their reliance on mycorrhizal fungi early in their life cycle. Tiny, dust-like orchid seeds have little room for food reserves that would otherwise nourish the embryo until the emergence of its first leaf. Because of this, orchids rely on a symbiotic relationship with fungi to develop from seed [4].

Mycorrhizal fungi are critical to understanding the distribution patterns of many plants, but they are especially important for orchids. Identifying the fungi that support orchid growth is important for determining where orchids grow, their population dynamics, and density [5]. In the identification of the fungi that are needed to sustain orchid populations, the most commonly used approach is to identify the fungi forming orchid mycorrhizae with mature plants (e.g., [6,7]), and to assume those fungi include the ones needed for seed germination and protocorm growth. Although this is clearly true for species that require specific fungi throughout their lives, orchids that associate with a wide range of orchid mycorrhizal fungi (OMF) once they become mature may require different or more specific fungi for germination and protocorm development. In these species, it is unclear how OMF identified from mature orchids relate to those that also support seed germination and protocorm development. Even if there is a difference between the OMF needed for early and later life stages, adult orchid roots may still retain the fungi that supported germination from seeds and early development [8,9,10]. Alternatively, adult orchid roots may be colonized by entirely different OMF, and fungi from adults may not support seed germination, as in *Cynorkis purpurea*, which appears to switch fungi during maturation [11].

Ventre Lespiaucq et al. [12] distinguished two categories of conditions that might drive change in OMF through time. First, changes in abiotic or biotic conditions, either seasonally or annually, could result in changes in the availability of fungi [13]. Alternatively, changes in the orchid, especially associated with ontogenetic changes, such as from a mycoheterotrophic protocorm to a photosynthetic seedling or adult [14], could produce changes in the association between the orchid and its mycorrhizal fungi that either require or support a shift in the OMF. When OMF change through time, Ventre Lespiaucq et al. [12] considered three types of change. First, through nested mycobiont gain or loss the OMF present at one time can be a subset of those present at another time. In this situation, the diversity of OMF can remain the same, increase, or decrease over time. Second, OMF present at one time can be replaced by completely different fungi. Third, there can be partial replacement, where diversity remains the same, but some OMF are replaced and others are retained.

Although different scenarios for changes in OMF have been identified, details of the changes that are known to occur are few and it is unclear how widespread any of the scenarios are across the orchid family [15]. In particular, the extent to which OMF change as orchids mature is not well known, largely because identifying the fungi needed by protocorms and early life history stages in which the orchids are very small often requires multiple years and is impractical in many study systems.

Switching OMF during ontogeny may be beneficial for many reasons. The ability to switch fungi may allow tolerance of varied environmental conditions or disturbance (e.g., [16]) or may support different nutritional needs and lower dependence on fungi as orchids mature [10,17]. An increased diversity of OMF with the onset of photosynthetic capabilities might be expected because the needs of the plant may change dramatically from fully mycoheterotrophic to partially mycoheterotrophic or mixotrophic, or even largely photosynthetic. It is also possible that switching fungi might be related to competition between adults for fungal resources [18,19]. Alternatively, the habitats where seed germination occurs may be short-lived, and different conditions, which might support different fungi, may prevail either seasonally or by the time the orchids reach maturity (e.g., [11]). Similar shifts have been reported in *Gastrodia elata* [20] and *Arundina graminifolia* [15].

One orchid in which specific fungal requirements for protocorm development and more general mycorrhizal associations with maturity have been identified is the temperate terrestrial orchid *Tipularia discolor* [10,21]; however, the drivers of the changes in the OMF during ontogeny remain unclear. *Tipularia discolor* (Pursh) Nutt. is a winter-green terrestrial orchid native to the Eastern United States [22]. The species associates with several species of *Tulasnella* fungi as an adult, but exhibits stricter specificity during the protocorm stage, which is the stage from germination until a leaf is produced [10]. Although many *Tulasnella* fungi have been cultured from roots of adult *T. discolor*, numerous attempts to isolate and culture the fungi from protocorms over the course of 20 or more years have all been unsuccessful, despite the presence of abundant pelotons. This suggests that if these fungi were present in adult orchid roots, then they would not be detected using culture-based techniques. McCormick et al. [10,23] found that all fungi associated with protocorms belonged to one of two distinct, closely related, but taxonomically undescribed, fungal taxa.

Protocorms and seedlings of *T. discolor* are consistently associated with decomposing wood [21], but mature *T. discolor* are frequently found on bare soils that have no obvious residual decomposing wood. Prior to recent molecular applications, the relationship between this orchid, its mycorrhizal fungi, and decomposing wood remained unclear. To obtain a broader sample of the fungi associated with protocorms, so we could ensure we were assessing the full diversity of protocorm fungi in adult orchid roots and substrates, we first obtained all published sequences from *T. discolor* protocorms from Genbank that were submitted by McCormick et al. [10,23].

We addressed questions related to changes in OMF using *T. discolor* as a model species: (1) Do mature plants retain protocorm mycorrhizal fungi into adulthood, or are protocorm and adult orchid mycorrhizal fungi mutually exclusive? (2) Are protocorm mycorrhizal fungi limited to areas with decomposing wood, which are the only places where protocorms have been found? (3) Does the distribution of protocorm mycorrhizal fungi in the soil limit the germination of *T. discolor* seeds? We hypothesized that: (1) mature plants would switch OMF between the protocorm and adult stages; (2) protocorm mycorrhizal fungi would only be found in decomposing wood and would be absent from other substrates; and (3) germinated *T. discolor* seeds would be associated with substrates where protocorm mycorrhizal fungi were abundant.

## 2. Results

**Figure 1:**
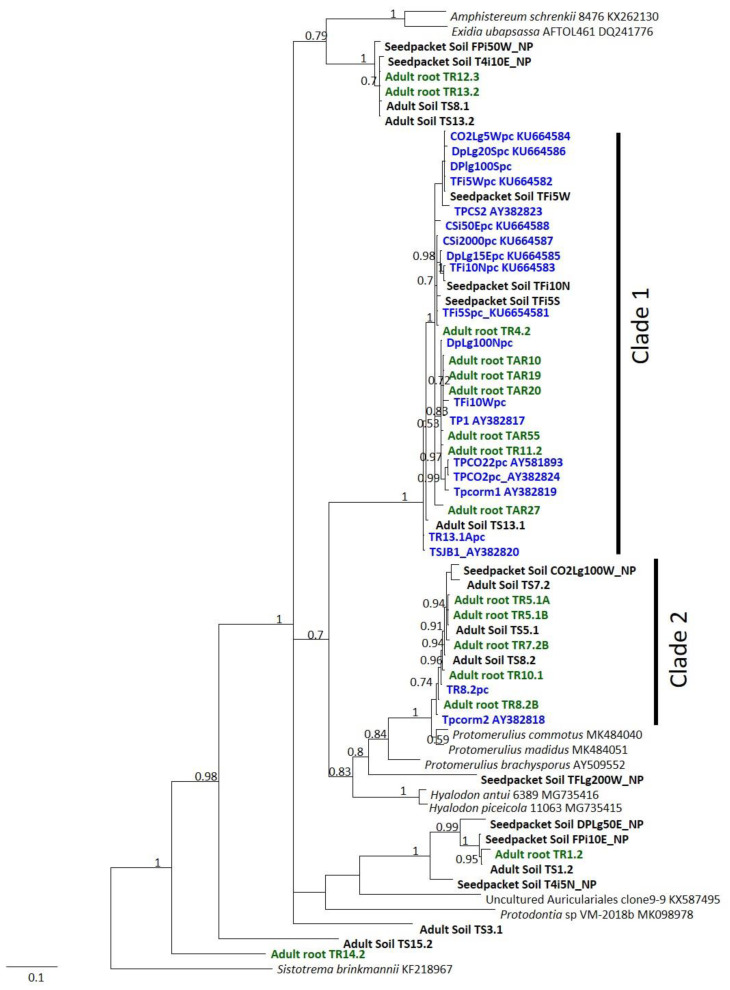
Bayesian phylogenetic tree based on ITS sequences showing Auriculariales fungi from protocorms (blue), adult roots (green), and soil samples (bold, black). Posterior probabilities ≥0.7 are given to show node support. The delimitation of the two clades of fungi that include protocorm fungi is indicated by vertical bars labeled Clade 1 and Clade 2. Labels for sequences from soil samples associated with *T. discolor* adult roots begin with “Adult soil”, followed by the sample number. Names of sequences from adult orchid roots begin “Adult root”, followed by the sample number. Names of soils adjacent to seed packets begin with “Seedpacket Soil”. Labels for soil samples adjacent to seed packets without protocorms end with “_NP”. Labels for sequences from protocorms in previous studies end with their Genbank accession number.

We found that all fungal sequences from protocorms belonged to the two clades (Figure 1) that were previously described by McCormick et al. [10]. Each of these two clades was well supported, with posterior probabilities of ≥0.98. More recent sequences in GenBank identified Clade 2 fungi as belonging to the *Protomerulius* genus in the order Auriculariales, most likely *Protomerulius madidus*. Fungi in Clade 1 also had *Protomerulius* fungi as the closest identified matches in Genbank, but phylogenetic analysis grouped them as a distinct clade within the Auriculariales. Fungi within these clades included those sequenced from protocorms, adult plant roots, and substrates. Other fungi in the Auriculariales and Agaricales were also sequenced from roots of adult plants and substrates. Results from this study add diversity to the wide range of *Tulasnella* fungi that were previously described from roots of *T. discolor* adults by McCormick et al. [10].

Positive PCR product was obtained from 69% of adult *T. discolor* roots using the two primer pairs that were designed to amplify fungi from both clades found in protocorms. Clade 1 fungus primers resulted in positive PCR product from 23% of adult roots, while Clade 2 fungus primers produced positive PCR product from 54% of adult roots. Four roots (8%) produced PCR product with primers for both clades of protocorm fungi. Both sets of protocorm fungus primers were similarly likely to produce PCR product from the roots of orchids growing with and without decomposing wood (logistic regression, substrate main effect, *p* = 0.20).

Positive PCR product was also obtained from 50% of substrates associated with adult roots using the two protocorm fungus primer pairs. Clade 1 primers resulted in positive PCR product from 21% of substrates, whereas Clade 2 fungus primers produced positive PCR product from 38% of substrates. Three substrates (9%) produced PCR product with primers for both clades of protocorm fungi. Both sets of primers were more likely to produce PCR product from decomposing wood than from soil substrates (logistic regression, substrate main effect, *p* = 0.001). DNA sequences were obtained from 16 adult roots and nine substrates appressed to adult roots. Twelve adult root sequences fell within the two clades of protocorm fungi (seven in Clade 1, five in Clade 2), as did sequences from four associated soils (one in Clade 1, three in Clade 2). We also obtained sequences from four substrates that were appressed to seed packets with protocorms, all four of which belonged to protocorm fungus clades (three in Clade 1, one in Clade 2), and eight substrates appressed to seed packets without protocorms, none of which belonged to a protocorm fungus clade. Six of these sequences belonged to the Auriculariales, but not to either clade of protocorm fungi (Figure 1), whereas two fell outside the Auriculariales and were not included in the phylogenetic tree.

**Figure 2:**
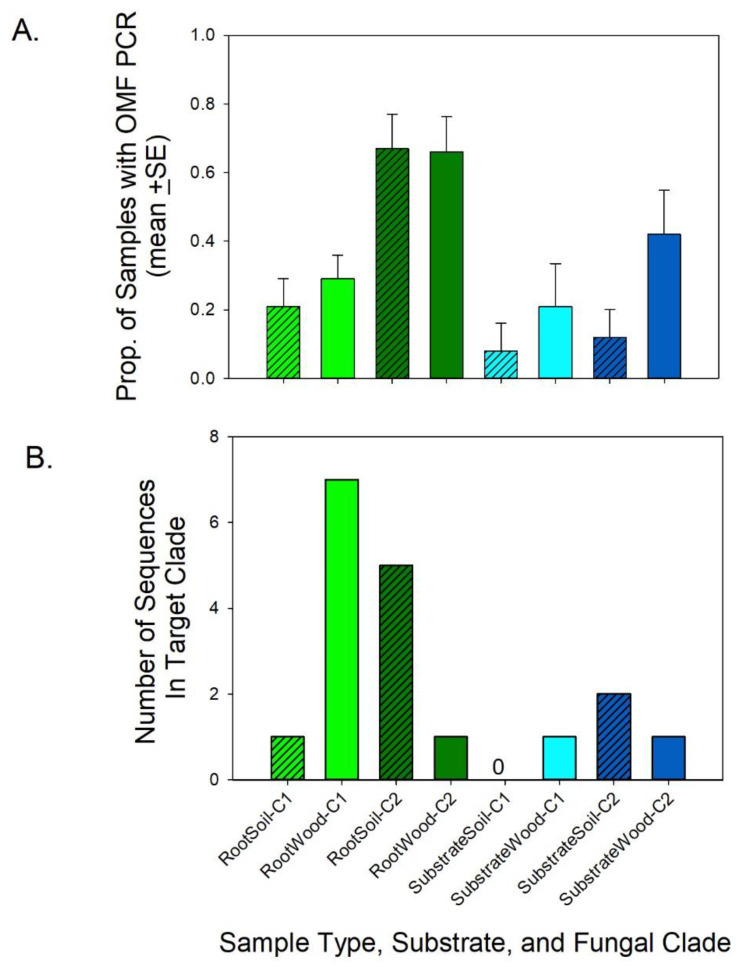
Prevalence of protocorm fungi. (**A**) Proportion of samples from roots (green bars) and substrates (blue bars) adjacent to roots of mature *T. discolor* plants growing in substrate rich in decomposing wood (solid bars) or bare soil (hatched bars) and (**B**) number of DNA sequences obtained from roots and substrates using PCR primers specific to Clade 1 (light green and light blue bars) and Clade 2 (dark green and dark blue bars). X-axis labels indicate the sample type (Root or Substrate), the substrate type (Wood or Soil), and the fungal clade presented (Clade 1 (C1) or 2 (C2)).

Fungi (regardless of source) that belonged to the two clades that contained protocorm fungi had different occurrence patterns in protocorms, adult plant roots, and substrates, but the pattern depended on whether amplification of PCR product or successful DNA sequencing was considered (Figure 2A,B). Only sequences were analyzed for protocorms. Clade 1 fungi were the most common fungi sequenced from protocorms and included sequences from 14 of the 15 sequences from protocorms in published studies and three of the four sequences from protocorms in this study. Clade 2 primers were more likely to produce PCR product from adult orchid roots (Clade 1: 12/52, Clade 2: 28/52) and from substrates (Clade 1: 7/34, Clade 2: 13/34) than Clade 1 primers. In contrast, the two primer sets were similarly likely to produce sequences from mature plant roots (Clade 1: 8/51, Clade 2: 6/51; Figure 2B). Only one substrate sequence associated with an adult root belonged to Clade 1 and three belonged to Clade 2.

Considering positive PCR products, adult plant roots were similarly likely to have positive PCR product from the two primers in wood and soil substrates (Clade 1: 19/51, Clade 2: 21/51; *p* = 0.5, Figure 2A). Roots of adult orchids that were growing in decomposing wood more frequently had Clade 1 protocorm fungi detected using sequences. However, based on the sequences obtained, many of the PCR products, especially those using the Clade 2 primers, may have belonged to taxa outside of the target clades. Although many of the substrate sequencing efforts using both primer sets failed because of poor sequence quality, likely attributed to a mix of PCR products, five of the Clade 2 sequences belonged to fungi outside of the target clades, but within the Auriculariales, while only one Clade 1 sequence did.

**Figure 3:**
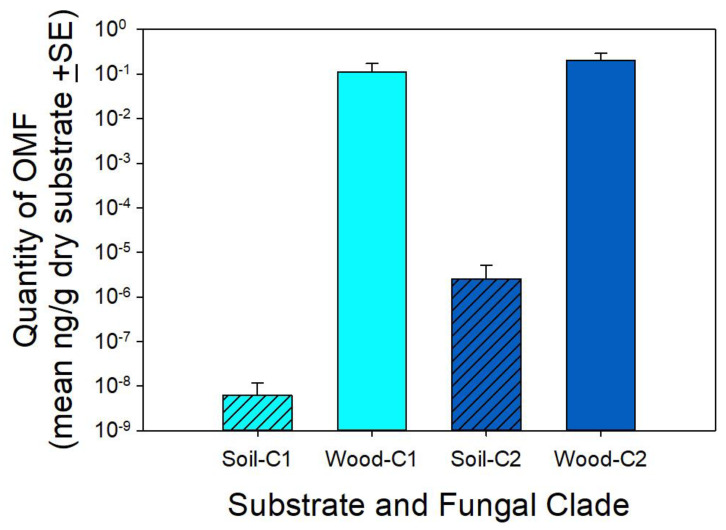
Abundance of the two clades of protocorm fungi in substrate rich in decomposing wood (solid bars) and soil (hatched bars) substrates. Clade 1 quantity is indicated by light blue bars and Clade 2 by dark blue bars. Note the log scale on the y-axis.

For the fungi associated with seed packets, seven of the 11 successful sequences from substrates that were associated with seed packets were outside of the two target clades. All seven were associated with seed packets that had no protocorms. The four sequences that fell within the target clades were all from soil associated with seed packets that had protocorms. Abundance estimates of fungal DNA in the substrate revealed stronger differences between wood and soil than was apparent from fungal presence alone. Both clades of protocorm fungi were significantly more abundant in decomposing wood than in soil (ANOVA; F-ratio = 9.5, *p* = 0.003; Figure 3) and results of the two primer sets were similar. Neither the primer set nor the interaction between the primer and substrate had a significant effect on measures of protocorm fungus abundance (both F-ratios < 0.9, both *p* > 0.3).

## 3. Discussion

We found that fungi that are found as pelotons in protocorms were retained in roots of mature plants, even when the plants were no longer growing in association with decomposing wood. This suggests that *T. discolor* increased mycorrhizal fungus diversity ontogenetically, rather than strictly switching fungi. The relationship between protocorm mycorrhizal fungi and adult plants is, however, not universal, because many mature individuals lacked detectable protocorm fungi. This is consistent with the scenario of nested symbiont turnover, as described earlier, through symbiont gain [12].

Roots of some adults retained the protocorm fungi after they were no longer associated with decomposing wood. In some instances, the adults that had protocorm fungi were growing in the vicinity of protocorms, indicating that the protocorm fungi were likely present and abundant in the substrate. In other instances, adults with protocorm fungi were growing in bare soil, where we had not observed protocorms. Roots of adult *T. discolor* generally have multiple OMF, which is why we used primers that were specific to the protocorm fungi to sequence them. In contrast, protocorms generally only have their protocorm fungus, and all original sequences of these fungi were made using general fungal primers [10]. This provides further evidence that the change in the presence of other OMF in adult roots is tied to ontogeny rather than being associated with substrate changes. Increases in fungal diversity associated with ontogeny may allow orchids to incorporate more diverse sources of fungal support, providing flexibility in response to changing environmental conditions (e.g., [16]) or changes in substrate that occur as the wood in which the fungus originally occurred was completely decomposed. It also suggests that the increased fungal diversity and/or altered composition may be related to the different nutritional needs and lower dependence on fungi as orchids mature and develop photosynthetic capacity [10,11,17]. Ontogenetic shifts could also be associated with limiting competition between protocorms and adults for fungal resources [19,24]. Alternatively, retaining protocorm fungi could enable the older and larger individuals to continue to benefit from the presence of protocorm fungi, as long as the decomposing wood provides a suitable substrate for the fungus. As substrate conditions change, the fungal community associated with the decomposing wood would change, potentially providing other fungi that could support the orchid as it matures. Similar shifts have been reported in *Gastrodia elata* [20] and *Arundina graminifolia* [15].

There is some, although very limited, evidence for each of the three scenarios for temporal change of fungal communities in orchids outlined by Ventre Lespiaucq et al. [12]. The majority of studies have found that, in some orchids, diverse OMF may initiate seed germination, but further development can only be accomplished by a few of those fungi [18,25,26]. The switch in OMF between the seed germination and protocorm stages, and later ontogenetic stages, represents a bottleneck of development that is regulated by relatively few fungi [14,17,25,27,28]. These were all examples of diversity increases from the protocorm stage to the mature orchid, and many found protocorm fungi also in adult roots.

Most previous studies of OMF associated with protocorms were conducted in the laboratory, where the changes were likely driven strictly by ontogeny, without attention to changing environmental conditions that would support orchids at different life stages. There have been a few field-based studies that found that OMF change at different developmental stages. Ran and Xu [29] found that *Gastrodia elata* associated with *Mycena osmundicola* during seed germination and protocorm development, whereas adults associated with *Armillaria mellea*, which, as Xu and Guo [30] found, inhibited seed germination. Claro et al. [31] found that two *Bipinnula* species had very specific associations with *Ceratobasidium* fungi at maturity, whereas those fungi were unable to effectively promote seed germination, and Bayman et al. [32] found that *Oeceoclades maculata*, an invasive species in Puerto Rico, could associate with a diverse array of saprotrophic fungi as an adult, but required an association with a *Psathyrella* species to germinate.

Studies of mature orchids have also detected either seasonal [33,34] or environment-driven [16,35] changes in the fungi that were not associated with ontogeny. We found [10] that the *Tulasnella* OMF associated with *T. discolor* adults reflected the available OMF in the soil where they were growing. The presence of multiple OMF in the soil explains why *T. discolor* growing near *Goodyera pubescens* or *Liparis liliifolia* had roots that contained the same OMF as the nearby species. These studies suggested that shifts in OMF in orchid roots reflect changes in fungal communities associated with altered habitat conditions and, thus, OMF availability.

In addition to the presence or absence of OMF in substrates in which orchids grow, results of this research provide additional evidence that the quantity of OMF may be an important component of orchid ecology. We found that *T. discolor* protocorm fungi were more abundant in substrates that included decomposing wood. This likely explains why protocorms are almost exclusively restricted to decomposing wood. McCormick et al. [23,36] found that seeds of four orchids, including *T. discolor*, only germinated when the mycorrhizal fungi they required were abundant. Rock-Blake et al. [37] and McCormick et al. [5] provided evidence that abundant OMF are critical for supporting mature orchid growth. If both clades of protocorm fungi decrease dramatically in abundance once wood completely decomposes, they may be present in the soil but at a very low abundance, which would decrease their potential to support adult plants where wood has fully decomposed.

Fungi in the Auriculariales generally, and in the genus *Protomerulius* specifically [38], are thought to be primarily saprotrophic fungi, and they are often dependent on decomposing wood. Although we do not know the ecological functions of the fungi that we identified in association with *T. discolor* protocorms, dependence of the fungi on decomposing wood could explain why abundant fungi belonging to both protocorm fungus clades were associated primarily with wood substrates. Fungi in the Auriculariales are not generally known to be orchid mycorrhizal fungi [39]. However, Umata [40] found that *Auricularia polytricha* formed mycorrhizas and stimulated seed germination in the achlorophyllous orchid *Erythrorchis ochobiensis*, and recent studies have identified other wood rot fungi as orchid mycorrhizae [41].

Clear limitations in the PCR method that we used to detect protocorm fungi in adult orchid roots and substrates influenced the results and subsequent interpretation. In particular, the specific primers that we used, especially those designed for Clade 2 fungi, occasionally amplified fungi from outside the target clade, as was apparent from the sequences we obtained. We only found this to happen when fungi from the target clades were likely to be almost entirely absent, suggesting that the primers preferentially amplified the target fungi, but that non-specific amplification occurred when target fungi were absent. This presents a clear problem for interpreting the PCR results, in which Clade 2 fungi were more commonly detected in substrates with decomposing wood. Because many fungi in the Auriculariales are also wood decomposers, many related groups of fungi, which would be more likely to be amplified with Clade 2 primers, would likely have been present in the decomposing wood samples. This may also have been responsible for the greater fungal abundance measured in wood substrates with Clade 2 primers for qPCR. A metabarcoding or metagenomics approach would have circumvented this problem, but would not provide quantitative comparisons, and metabarcoding techniques were not widely available or cost-effective when this study was conducted. Nevertheless, the frequency with which fungi in each clade were identified in sequences verified that protocorm fungi were present in adult orchid roots, although perhaps less commonly than suggested by positive PCR product.

The frequency pattern based on sequences may also suggest that substrate changes affected fungal shifts. This differed from the interpretation of PCR product alone. It is likely that both ontogenetic and environmental shifts play a role in determining the colonization of adult orchid roots by mycorrhizal fungal communities. It is also worth noting that the occurrence of protocorm fungi in sampled *T. discolor* adults is likely an underestimate of their actual occurrence. We only analyzed partial root systems. Larger roots that we divided into two sometimes tested positive in one portion and not the other, suggesting that the protocorm fungi may be localized and so could have been missed by analyzing only part of the root system.

Alternatively, there were root and substrate samples with positive PCR product, but for which we did not obtain clean sequences. Most of the sequences we obtained belonged to the target fungal clades. We only obtained sequences outside the target clades from a few samples, but the presence of other fungi that were amplified by our specific primers might have produced a bias against sequencing success in roots and substrates that had more diverse fungi within the Auriculariales, because they would produce mixed sequences. This might have driven the decrease in the number of Clade 2 sequences that we obtained from roots and substrate samples associated with decomposing wood, compared to those associated with soil. If Auriculariales fungi are predominantly wood decomposers, then they could be more diverse in the wood-associated samples and produce more mixed and unreadable sequences. Again, this could have been alleviated by using metabarcoding.

The non-specific amplification by specific primers likely also affected the estimates of Clade 2 fungal abundance obtained from qPCR. In particular, the greater abundance of Clade 2 fungi in decomposing wood substrates may have been attributed to non-specific amplification. However, there was considerably less non-specific amplification with the primers for Clade 1 fungi, suggesting that the increased abundance of that fungal clade in decomposing wood substrate is more believable.

Despite the limitations and technical issues described above, our data demonstrate that the locations where protocorms were found, i.e., sites with decomposing wood, were more likely to have protocorm fungi, and that decomposing wood had more abundant protocorm fungi than soil. Positive PCR suggested that the roots of mature orchids were equally likely to have protocorm fungi as associates if they were growing in decomposing wood or in soil. This might suggest that protocorm fungi are protected within the orchid roots, as has been suggested by Selosse [3]. Alternatively, based on the frequency of successful sequences that belonged to the two target protocorm clades, Clade 1 fungi may have been more common in the roots of adult orchids that were associated with wood than in those growing in soil, whereas Clade 2 fungi had the reverse relationship. Regardless of relative frequencies, we found that protocorm fungi were, at least sometimes, retained in the roots of mature orchids and that the diverse OMF in the roots of mature *T. discolor* represent an ontogenetic increase in diversity. The study leaves open the possibility of an additional role for environmental change, because short-lived seed germination conditions (i.e., decomposing wood) change, in determining mycorrhizal fungus composition. These findings emphasize the importance of determining the fungi and conditions needed for seed germination, when attempting to develop orchid conservation and restoration programs.

## 4. Materials and Methods

We extracted DNA from peloton-containing sections of two protocorms from the seed packets used in the McCormick et al. [23] study and from two naturally-occurring protocorms that were found in association with locations where we sampled roots of adults growing in decomposing wood. We used DNEasy plant Mini-kits (Qiagen, LLC, Germantown, MD, USA) and amplified the ITS region of mycorrhizal fungi using the fungus-specific primers ITS1F/ITS4 [42,43]. Amplification reactions of 25 μL were carried out with a final concentration of 0.5 μM each primer, 0.1 μL BSA, and 50% Red Mix Plus PCR Master Mix (PGC Scientifics, Fredrick, Maryland, USA). An additional 0.65 μL of 25 mM MgCl_2_ was added to each reaction. Amplifications consisted of 35 cycles in an MJ Research DNA Engine and employed a 3 min initial denaturation at 94 °C before and elongation for 10 min at 72 °C after thermocycling. Each cycle consisted of a 30 s denaturation at 94 °C, followed by an annealing step of 30 s at 54 °C and elongation for 30 s at 72 °C. Reactions with no template DNA were performed with each amplification to ensure no contaminants were present. Additional PCR reactions with *Tulasnella*-specific primers (ITS5/ITS4-tul) were conducted to ensure *Tulasnella* fungi, which would not be amplified by ITS1F/ITS4, were absent, but no PCR products were obtained with the *Tulasnella*-specific primers. PCR product from each successful reaction was subjected to DNA sequencing using Big Dye v. 3.1 chemistry and analyzed on an ABI 3100 sequencer (ABI, Inc., Foster City, CA, USA).

After compiling sequences of mycorrhizal fungi in protocorms, we examined the presence and abundance of mycorrhizal fungi that belonged to the protocorm-associated taxa in roots of mature *T. discolor* that were associated with decomposing wood or bare soil. In the winter of 2006–2007 we collected one root from each of 37 mature *T. discolor* plants that were associated either with decomposing wood (*n* = 19) or bare soil (*n* = 18). To ensure plants were as widely distributed as possible, we searched each forested area of the 1076-hectare Smithsonian Environmental Research Center (SERC) property until we found *T. discolor* plants associated with decomposing wood. These sites included large decomposing tree trunks, where the woody tissue was clearly visible and constituted the majority of the substrate. In this advanced stage of decay, no effort was made to determine tree species, but all were hardwoods. Previous studies [21] demonstrated that *T. discolor* protocorms develop in decomposing wood of multiple hardwood species. We then searched the surrounding area for plants associated with bare soil (i.e., soil without any obvious pieces of organic material). Soils in the entire sampling area are sandy and fine sandy loams in the Collington, Monmouth, and Donlonton series [44]. We washed the outside of each collected root and protocorm with dish soap and dissected sections of root and protocorms with visible fungal pelotons for DNA extraction using DNEasy Plant Mini Kits (Qiagen). Larger roots with multiple areas that contained pelotons were divided into multiple DNA extractions to ensure all colonizing mycorrhizal fungi were considered.

We also analyzed roots collected from mature *T. discolor* that were associated with decomposing wood (*n* = 8) or bare soil (*n* = 8) that had been collected haphazardly around SERC property in 2003 and stored at −20 in CTAB buffer. For each root in this collection, we used the same procedures described above.

To first verify that our specific primers successfully amplified protocorm-associated fungi from the soil, we analyzed DNA from soils associated with the 10 seed packets for which published protocorm sequences were available [23], in addition to those associated with 40 seed packets without protocorms. These DNA extracts were previously used to quantify protocorm fungi in soils using semi-quantitative PCR [35]. We used the DNA extracts from soil adjacent to the 10 seed packets with protocorms and an additional 10 soil extracts associated with seed packets without protocorms to attempt to sequence *T. discolor* protocorm fungi, as below.

At the time of the 2006–7 adult *T. discolor* root collection, we also collected samples of ~2 g of soil adhering to and immediately adjacent to each sampled root and placed them into 20 mL vials. For the 2003 roots, we had no corresponding soil samples. Soils from 2006–7 were kept on ice until they were returned to the lab where they were frozen at −80 °C. The frozen samples were lyophilized, and ground with a mortar and pestle. We extracted DNA from one 0.25 g subsample of each ground soil sample using Fast Spin DNA kits for soil (Qbiogene, Irvine, CA, USA). We then amplified DNA obtained from roots and soils using PCR primers that were specific to the two clades of fungi that support *T. discolor* seed germination and protocorm growth (TipC2F-Tip14R and TipC1F-Tip14R; Table 1; [23]).

Amplification and sequencing were carried out for each primer set using the same cycle conditions as for ITS1F/ITS4, except with an annealing temperature of 59 °C. The presence of protocorm fungi in substrates was determined in two ways. First, the presence of PCR product for each of the two specific primer sets was noted as the presence of a band of the expected size on a 1% agarose gel. Second, we attempted to sequence all positive PCR products, using the same methods as for protocorm fungi, but with the two sets of specific primers. Obtained sequences were compared to sequences from *T. discolor* protocorms to verify that the amplified fungi belonged to the target taxa. Adult plant roots were considered to contain the protocorm fungi if a sequence obtained from the roots was at least 97% identical to a protocorm fungus sequence and it was placed within one of the two protocorm fungus clades. In cases in which large roots were divided, if one portion of the root produced positive PCR product, then we considered the entire root positive for the presence of protocorm fungi.

In addition to determining whether protocorm fungi were present, we also quantified the amount of protocorm fungus DNA in the substrate samples described above by subjecting substrate DNA extracts to quantitative real-time PCR on a Viia 7 (Applied Biosystems, Inc., Foster City, CA, USA), using the two protocorm-fungus specific primer sets in separate reactions. Reactions were carried out using 40 ng DNA in 20 µL reactions containing 10 µL IQ-SYBR Green MasterMix (BioRad, Hercules, CA, USA), 1 µL each primer (10 mM), 0.1 uL BSA, and sterile water as the remainder. Cycles consisted of an initial denaturation at 96 °C for 5 min, followed by 41 cycles of 15 s at 96 °C, 30 s at 60 °C, and 30 s at 72 °C. Note that a higher annealing temperature was used for these reactions, compared to sequencing from adult orchid roots, to increase stringency. A melting curve was carried out at the end of the final cycle to verify specificity of the reaction. Five 1:10 serially diluted quantitative standards were generated using an extract from a protocorm that was verified to contain the target fungus. Different protocorms were used for the two primer sets because each protocorm had only one of the two protocorm fungi.

To place the sequences from protocorms, adult roots, and substrates in a phylogenetic context, we conducted a BLAST search in GenBank using our sequences and extracted the two most similar sequences with at least 80% coverage. The ITS sequences we generated from protocorms and substrates, plus the GenBank sequences, were aligned using MAFFT, implemented in Geneious Prime (v.2020.2.5, BioMatters Ltd., Auckland, New Zealand), and adjusted manually. We then used Mr Bayes to generate a phylogenetic tree, using a sequence from *Sistotrema brinkmannii* (KF218967) as an outgroup, as per Spirin et al. [45]. We placed sequences from substrates, those deposited in GenBank from previous studies, and those from protocorms in a phylogenetic tree to determine the correspondence between these groups of sequences and to place those sequences in a taxonomic framework. Confidence in the tree nodes was assessed using posterior probabilities. Based on BLAST searches, we also downloaded sequences of other species within the Auriculariales as outgroups to various clades and to root the phylogenetic tree. Adult root and substrate sequences that fell within one of the two clades of protocorm fungi and were at least 97% similar to fungi within the clade were considered to be protocorm fungi, whereas fungi outside of these clades were considered distinct.

### Statistical Analysis

We compared the probability of adult *T. discolor* roots containing protocorm fungi using logistic regressions (Systat 11 for Windows) on whether positive PCR product and sequences were obtained from each sample, with substrate as the main effect. We conducted separate analyses for the root and substrate samples. The quantity of protocorm fungi detected in the substrates was compared using ANOVA, with substrate as a fixed effect. For the root samples, we first compared the two sampling times (2003 vs. 2006–7) by including sample time as an independent variable in the ANOVA, but the difference between samples collected at these two times was not significant (for year main effect and all interactions, all *p* ≥ 0.45, all F ≤ 0.45), so samples from the two years were combined.

## Figures and Tables

**Table 1:** PCR primers used in this study. TipR is the reverse primer for both forward primers. Reference for all is McCormick et al. [23].

Primer	Target Clade	Sequence	tm
TipC1F	*Tipularia* protocorm clade 1	TGCGAATGTGTCCCTCACAC	59
TipC2F	*Tipularia* protocorm clade 2	CGTGTTCATCATCCTCACACCT	59
TipR	*Tipularia* protocorm fungi	TGCATTTCGAGACGAGCCG	58

## Data Availability

Data are available from the authors (M.M., D.W.). DNA sequences are available from Genbank (accessions MZ383153-MZ383192).
